# Low-burden preventative digital mental health interventions for first-year college students: A pilot feasibility microrandomized trial

**DOI:** 10.1016/j.invent.2026.100912

**Published:** 2026-01-26

**Authors:** Adam G. Horwitz, Shriya Anand, Megan Chen, Kaitlyn McCarthy, Stephen M. Schueller, Maureen Walton, Srijan Sen, Cheryl A. King

**Affiliations:** a University of Michigan Medical School, Department of Psychiatry, 4250 Plymouth Rd., Ann Arbor, MI, 48105, United States of America; b Saint Louis University, Department of Psychology, 3700 Lindell Blvd., Saint Louis, MO, 63108, United States of America; c University of California, Irvine, Department of Psychological Science, 4201 Social and Behavioral Sciences Gateway, Irvine, CA, 92697, United States of America; d University of Michigan, Eisenberg Family Depression Center, 4250 Plymouth Rd., Ann Arbor, MI, 48105, United States of America

**Keywords:** Digital mental health, Personalized feedback, Depression, Text messaging, Stress, College students

## Abstract

**Introduction:**

Mental health problems among college students have increased significantly and barriers to care contribute to a substantial treatment gap. Digital mental health interventions (DMHIs) show promise for overcoming barriers, but engagement with DMHIs is challenging, underscoring the need for low-burden strategies.

**Objective:**

This pilot trial evaluated the feasibility and acceptability of a six-week, low-burden, preventative DMHI that delivered supportive text messages and personalized feedback (PF) to first-semester college students.

**Method:**

Students (*N* = 120, 64% women, 55% non-Hispanic White) who had mild-to-moderate depressive symptoms (PHQ-9 scores between 5 and 14) and were not engaged in formal mental health care were randomized to intervention (*n* = 90) or assessment-only (*n* = 30) conditions. Those in the intervention condition received a weekly PF report and/or supportive text messages at random intervals as part of an embedded micro-randomized trial (MRT). Primary outcomes were feasibility and acceptability of the intervention components. Exploratory analyses examined 1) clinical outcomes after six weeks for the intervention and assessment-only conditions, and 2) weekly clinical outcomes within the intervention group based on the MRT.

**Results:**

The trial demonstrated high feasibility (95% enrollment; 87% retention) and strong intervention acceptability, especially for PF and assessment components. Exploratory analyses did not reveal consistent patterns in between- and within-group comparisons.

**Conclusions:**

Low-burden strategies for assessment and intervention are feasible and acceptable to first-year college students at risk for depression. There is significant potential for integrating these lower-intensity strategies into a full-scale trial that adaptively delivers higher-intensity DMHIs and/or integrate human-delivered components in response to needs over time.

## Highlights

• 95% of eligible participants enrolled in the trial; 87% retention over six weeks
• 77% agreed personalized feedback reports provided valuable mental health insights.
• Over 90% agreed daily and weekly surveys helped to promote self-reflection.
• Supportive text messages were seen as less useful; only 46% found them helpful.
• Scalable DMHI model with potential for integration with higher-intensity supports

## 1 Introduction

The prevalence of mental health problems, such as depression, anxiety, and suicidal ideation, significantly increased among U.S. college students from 2007 to 2017 (Lipson et al., 2019) and remained elevated through 2024 (Healthy Minds Network, 2025). Elevated rates of depression and anxiety have also been observed for college students internationally (e.g., Auerbach et al., 2018; Li et al., 2022). The transition to college is characterized by numerous stressors, including new academic pressures, living arrangements, and interpersonal relationships and contexts (e.g., Byrd and McKinney, 2012; Emmerton et al., 2024), with intensive longitudinal studies demonstrating significant increases in stress and mental health symptoms during the first semester (e.g., Garett et al., 2017; Rogers et al., 2018). Additionally, most individuals start college in their late teens and early twenties, a time period when risk for onset of mood disorders is greatest (e.g., Beck et al., 2024; de Girolamo et al., 2012). Depressive episodes during this period can have significant consequences on physical health, social functioning, and academic performance (e.g., Bruffaerts et al., 2018; Leow et al., 2025; Wickrama et al., 2009).

While many evidence-based treatments have been developed for depression and mood problems, only 1 in 4 US adults with depression receive minimally adequate treatment (Thornicroft et al., 2017). In a longitudinal study of college students, over 60% of those with a mental health problem at baseline had a mental health problem two years later, and fewer than half of those with a mental health problem at both timepoints received treatment (Zivin et al., 2009). Internal barriers likely play a role with many individuals unaware of, or unable to name, their depressive symptoms until their distress, symptoms, or impaired functioning are severe (e.g., Epstein et al., 2010). Further, many college students report a low perceived need for care, even when exhibiting significant symptoms (e.g., Horwitz et al., 2020; Pei et al., 2024). Lack of time, self-reliance, and stigma are also impactful internal barriers to seeking and engaging in care among college students (e.g., Czyz et al., 2013; Lui et al., 2024). External barriers (e.g., availability of services) also contribute to lack of mental health care, as increased demand for mental health services has left college counseling centers overwhelmed, resulting in long waitlists or limited services (Xiao et al., 2017), leaving many students in need (Auerbach et al., 2016).

Digital mental health interventions (DMHIs) demonstrate promise for overcoming barriers to traditional mental health care by delivering interventions directly to individuals through their phones or computers (e.g., Lattie et al., 2019a; Philippe et al., 2022; Taylor et al., 2024). As a result, many universities have incorporated technology-enabled mental health resources to expand reach of their services (e.g., Lattie et al., 2019b). A review by Bergin et al. (2020) demonstrated the promise of preventive DMHIs for both primary (i.e., supporting mental health before problems begin) and secondary (i.e., detecting and responding to early signs of distress) prevention, while also noting a need for co-design processes that allow for greater adoption and sustained use. Indeed, high dropout rates and low utilization of mobile and web-based treatments present significant challenges (e.g., Borghouts et al., 2021; Mohr et al., 2013). In particular, college students report poor user interface, lack of accountability, and desire for more human support as barriers to sustained engagement with DMHIs (e.g., Bautista and Schueller, 2023; Braun et al., 2023; McCarthy and Horwitz, 2025; Melcher et al., 2022).

Lack of sustained engagement with DMHIs may be explained by a dynamic interplay of personal characteristics, intervention requirements, and technology features, whereby treatment workload exceeds personal capacity (e.g., Cross and Alvarez-Jimenez, 2024). As such, there may be advantages to DMHIs offering “low-burden” approaches that are less time-intensive, less technologically complex, and don't overwhelm an individual's workload capacity. Personalized feedback (PF) interventions are low-cost and low-intensity, often including just a brief screen or survey for a condition of interest, a written or visual summary of their score (often in relation to a standardized category or group norm), and available resources (e.g., Peter et al., 2019; Saxton et al., 2021). PF interventions facilitate behavioral changes by targeting constructs associated with the health belief model, including perceived susceptibility to a condition, perceived benefits of a behavior change, perceived barriers to making a behavior change, and self-efficacy to make a behavior change (Champion and Skinner, 2008). PF interventions have demonstrated effectiveness targeting numerous health behaviors, including substance use, gambling, weight loss, and depression (e.g., Geisner et al., 2006; Neighbors et al., 2004; Neighbors et al., 2015; Sherrington et al., 2016), and may be especially engaging for college students who are not receiving formal care, but are experiencing clinically significant symptoms (Horwitz et al., 2022). In recent years, PF interventions have been successfully adapted for mobile platforms to provide regular, ongoing feedback, rather than a single time-point (e.g., Rabbi et al., 2015; Witkiewitz et al., 2014).

Text-messaging interventions, which involve sending one-way supportive messages that encourage adaptive strategies for coping with stress or symptoms, also demonstrate promise as a low-burden approach to modestly improve clinical outcomes (e.g., Agyapong et al., 2022; Senanayake et al., 2019). Expected participant effort may vary across text-messaging interventions, as some may seek to promote behavioral changes (e.g., behavioral activation) whereas others may simply provide messages of empathy, support, or encouragement. In either case, text messages are less technically complex compared to most web-based or mobile applications, and individuals can flexibly apply when they might attempt a suggested strategy to their own schedule. Importantly, the impact of messages and PF can be moderated by state-level factors, such as recent mood, activity, or sleep (e.g., NeCamp et al., 2020), suggesting a need for adaptive interventions that can provide text messages or PF only when it is expected to have a positive impact for the recipient. Experimental methods, such as microrandomized trials (MRTs), repeatedly randomize participants to different intervention conditions and can be extremely valuable for determining whether, or under what conditions, to deliver a digital intervention (e.g., Klasnja et al., 2015; Qian et al., 2022).

Taken together, there are opportunities for low-burden DMHIs that incorporate PF and text messaging to help individuals increase their awareness of symptoms and their self-efficacy to cope with distress, before symptoms become severe and require higher levels of care. This is especially needed considering the long-term persistence of symptoms and limited utilization of mental health services among college students (Ballester et al., 2022; e.g., Zivin et al., 2009). The primary aim of this pilot clinical trial was to evaluate the feasibility and acceptability of a low-burden, preventative DMHI that provided supportive text messaging and PF over a six-week period to first-semester college students who reported mild-to-moderate depressive symptoms and no formal mental health treatment. Exploratory aims included: 1) examining range and variability of clinical outcomes (i.e., depression, perceived stress) for the intervention and assessment-only groups after six weeks, and 2) within the intervention group, examining the results of an embedded MRT comparing the relative within-person effects of receiving or not receiving PF or text messages on proximal outcomes.

## 2 Method

This pilot RCT was conducted between October 1, 2024 and November 26, 2024. Informed written consent was obtained for participants and all procedures were approved by the University of Michigan Medical School Institutional Review Board (HUM00257547). This study report adheres to the Consolidated Standards of Reporting Trials (CONSORT; Schulz et al., 2010) reporting guidelines. The clinical trial was pre-registered on clinicaltrials.gov (NCT06583096).

### 2.1 Setting and participants

First-year college students were recruited, consented, and screened from a Midwestern University's flagship and satellite campuses. Inclusion criteria included being at least 17 years old and endorsing mild-to-moderate depressive symptoms (PHQ-9 total scores ranging from 5 to 14). Students who were receiving ongoing professional counseling or psychotherapy services at the time of screening were excluded. Data were collected via Qualtrics surveys delivered via e-mail (screen/baseline) and text messages (weekly surveys over six weeks).

### 2.2 Procedures

**Fig. 1:**
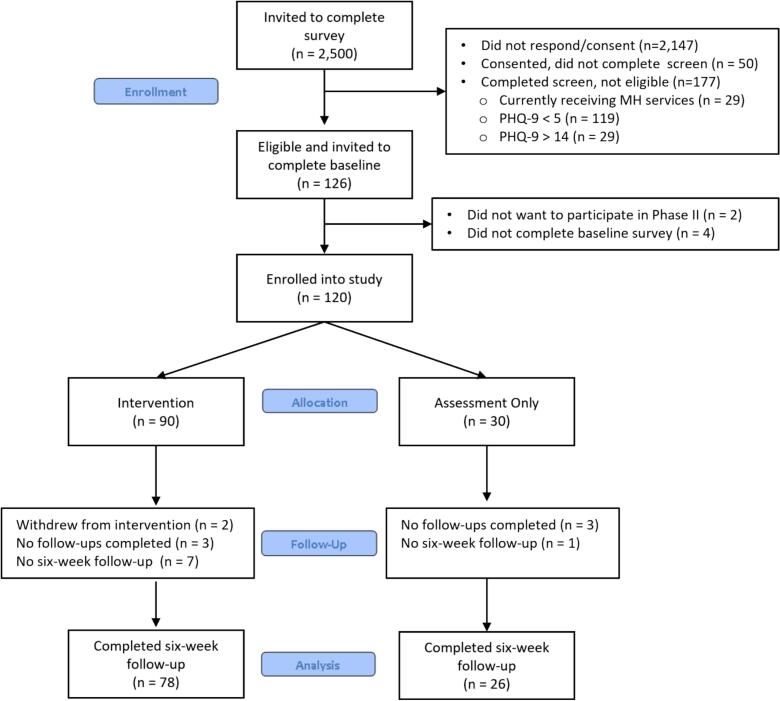
CONSORT flow diagram.

The registrar's office at two campuses provided limited data (i.e., first name, last name, email address) of first-year students (2000 at flagship, 500 at satellite) to the study team, which was stored in a secure HIPAA-compliant folder. Students were invited via e-mail during the fifth week of the Fall semester to complete an 8-minute mental health screen via Qualtrics, with two reminder e-mails sent three days apart for those who did not complete the consent and/or survey. Consent was provided by 353 (14.1%) students and 303 (85.8%) completed the full screen (please see Fig. 1 for CONSORT Flow). While participation rates were similar for screening across campuses, participants from the satellite campus were significantly more likely to be excluded from the intervention due to engagement in formal therapy or counseling (23.8% of those screened at satellite versus 5.8% of those screened at flagship)—these differences may be explained by students at the satellite campus retaining continuity in their living situation and being able to continue to engage with established providers, whereas flagship students were more likely to have moved away from their hometowns. Students were informed in the consent form that those invited into phase 2 of the study would receive weekly surveys over the next six weeks, with some participants receiving daily 1-minute surveys, text messages offering tips/strategies for managing stress, and PF reports regarding their survey responses.

Students were first randomized via parallel assignment (3:1) to either the intervention condition or an assessment-only condition in blocks of 20 (i.e., 15:5) to ensure balance. Among students assigned to the intervention condition, a microrandomized trial (MRT) design was used, whereby students were randomized each week, for six weeks, to receive or not receive (2:1) personalized feedback reports and/or text messages. Because randomization occurred weekly and independently for the two intervention components (personalized feedback, text messages), participants could receive both components, only one component, or neither component in a given week, and participants varied with respect to the number of times they received personalized feedback or text messages over the six week study. The greater allocations to the intervention group (3:1) and to the intervention components (2:1) were intended to increase available data for within-group MRT comparisons while balancing participant preferences for less frequency (McCarthy and Horwitz, 2025). Randomization sequences (i.e., yes/no to feedback/texts each week) were assigned to specific IDs, which were sequentially allocated to participants who completed the baseline survey.

Participants who completed the screen were enrolled in a raffle to win one of ten $50 digital Visa cards. Baseline participants were compensated with a $20 digital Visa card for completing the additional baseline surveys following the screen. Participants were compensated with $10 for each completed weekly 10-minute ‘Sunday survey’ during the first five weeks of the study and $25 for completing the final 20–25-minute follow-up assessment given on the sixth and final week. Participants in the intervention condition earned $1 for each daily 1-minute survey they completed over the course of six weeks. Accumulated funds were added to the balance of their digital Visa card weekly.

### 2.3 Study conditions

#### 2.3.1 Intervention condition

##### 2.3.1.1 Personalized feedback reports

**Fig. 2:**
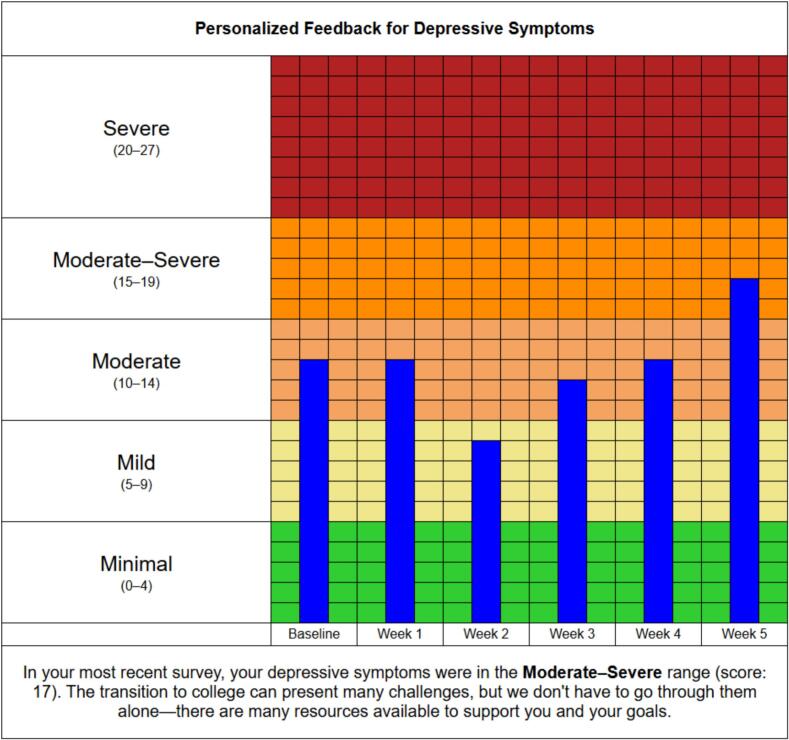
Sample personalized feedback graph.

Participants were randomized (2:1) every week for six weeks to receive (or not receive) a text message at 2 pm on Mondays containing a link to a PF report (“If you're interested, here's a graphical summary of your recent survey responses: [url link]”). The PF webpage provided bar graphs depicting scores of depression, anxiety, perceived stress, and flourishing, with columns (e.g., baseline, week 1, week 2, etc.) that populated as the study progressed (see Fig. 2) to demonstrate changes over time. Graphs were color-coded to depict severity levels of the measures (e.g., mild, moderate, severe). A brief summary indicating current severity level and a supportive statement [e.g., “In your most recent survey, your stress level was in the **Moderate** range (score: 8). It can take some time before we feel like we have a handle on all the new demands of college life, see whether this is something that improves with time or whether the difficulty increases,”] were also provided at the bottom of each graph. A final summary statement describing the current ratings from the four measures, along with links to available campus mental health resources, were featured at the bottom of the page.

##### 2.3.1.2 Supportive text messages

Participants were randomized (2:1) every week for six weeks to receive text messages, with 50:50 daily-level randomizations to receive a message (when randomized to receive messages that week). A bank of 25 messages was developed by the study team, informed by qualitative interviews (McCarthy and Horwitz, 2025), and feedback from students. Supportive messages covered a range of topics, including time management, self-care, coping with stress, and social engagement. Messages were randomly pulled from the message bank for each participant without repetition, generating unique sequences for each participant. Some messages were health promotive and did not assume underlying stress (e.g., “In the past week, what did someone else do or say to you that you are grateful for? Reflect on that feeling.”) whereas others were tailored for prevention (e.g., “Being resourceful is a sign of intelligence--asking for help from others when you're struggling is a strength.”). Some messages featured links to articles or videos (e.g., “Goals are great motivators… until we reach them. Using your values as motivators is key for sustainability. This video describes the difference between goals and values in greater detail: [url link]”). Messages were delivered at 11 am.

##### 2.3.1.3 Daily assessments

Participants received a daily text message prompt at 7 pm to complete a one-minute, five-item survey, which included a question about their mood, a question about their interest in daily activities, a question about satisfaction with social relationships, a question about emotional awareness, and a question about confidence to cope with life stress.

#### 2.3.2 Assessment-only condition

Participants in the assessment-only condition completed the same weekly surveys as those in the intervention condition, but did not receive daily surveys, personalized feedback reports, or supportive text messages. The closing page for completed surveys included a message acknowledging that the survey included questions regarding mental and emotional health, and provided crisis contacts and weblinks to mental health resources on their campus.

### 2.4 Outcomes

#### 2.4.1 Feasibility and acceptability (primary)

Feasibility of the trial procedures and intervention was operationalized as follows: percentage of eligible participants who agreed to participate in phase 2 of the study (i.e., clinical trial), percentage of follow-up assessments completed, participant retention through the final survey, percentage of PF reports viewed by participants, percentage of participants who viewed a PF report at least once. Acceptability was assessed by participant responses to satisfaction questions about the overall intervention and specific components (e.g., surveys, supportive text messages, PF reports) from the final weekly survey.

#### 2.4.2 Clinical outcomes (exploratory)

Participants in the intervention condition were compared to the assessment-only condition with respect to changes from baseline to the sixth weekly follow-up survey on measures of depression, anxiety, perceived stress, flourishing, coping self-efficacy, emotional awareness, loneliness, and help-seeking. For those in the intervention condition, within-person differences were examined for proximal end-of-week clinical outcomes (over six weeks) based on randomization to receive or not receive an intervention component during a particular week.

### 2.5 Measures

#### 2.5.1 Demographics

Participants were asked to report their age, gender identity, race/ethnicity, sexual orientation, and campus affiliation. Participants were able to check all that apply from a range of responses for gender identity, race/ethnicity, and sexual orientation; responses were coded into mutually exclusive categories. Gender identity included: cisgender man, cisgender woman, and transgender or non-binary. Race/ethnicity included: non-Hispanic White, Black, Hispanic, Asian, Multi-racial, and Other Race. Sexual orientation included: heterosexual, bisexual or pansexual, mostly heterosexual, and gay, lesbian, queer, or other sexual orientation.

#### 2.5.2 Depression

The Patient Health Questionnaire-9 (PHQ-9; Kroenke et al., 2001) is a nine-item scale that assesses the frequency of difficulties associated with the nine DSM-5 symptoms of depression. At the baseline assessment, participants responded regarding symptoms over the past two weeks, whereas subsequent assessments inquired about symptoms over the past week. Items are rated on a 4-point Likert scale ranging from 0 (*not at all*) to 3 (*nearly every day*), with total scores ranging from 0 to 27. Severity ratings include: *minimal* (0–4), *mild* (5–9), *moderate* (10–14), *moderately severe* (15–19), and *severe* (20+).

#### 2.5.3 Anxiety

The Generalized Anxiety Disorder-7 (GAD-7; Spitzer et al., 2006) is a seven-item scale that assesses the frequency of difficulties with seven anxiety symptoms associated with generalized anxiety disorder. At the baseline assessment, individuals responded regarding symptoms over the past two weeks, whereas subsequent assessments inquired about symptoms over the past week. Items are rated on a 4-point Likert scale ranging from 0 (*not at all*) to 3 (*nearly every day*), with total scores ranging from 0 to 21. Severity ratings include: *minimal* (0–4), *mild* (5–9), *moderate* (10–14), and *severe* (15+).

#### 2.5.4 Perceived stress

The Perceived Stress Scale (PSS; Cohen, 1988; Cohen et al., 1983) assesses frequency of thoughts and feelings associated with stress. The ten-item version was administered at baseline (asking about past month) and final assessments (asking about past week), whereas the 4-item short form was used to assess past-week stress as part of weekly Sunday surveys. Items on the PSS are rated on a 5-point Likert scale ranging from 0 (*never*) to 4 (*very often*), with total scores ranging from 0 to 40, with higher scores indicating greater perceived stress. PF reports provided scores on the PSS-4, with total scores ranging from 0 to 16 and featuring the following severity ratings: low stress (0–5), moderate stress (6–10), high stress (11–16).

#### 2.5.5 Flourishing

The Flourishing Scale (Diener et al., 2010) is a brief 8-item summary measure of self-perceived success in typically important domains of life such as relationships, self-esteem, purpose, and optimism. Items on the scale are rated on a 7-point Likert scale ranging from 1 (*strongly disagree*) to 7 (*strongly agree*), with totals ranging from 8 to 56 and higher scores suggesting greater psychological well-being. PF reports provided scores with the following ratings: Struggling (8–40), Managing (41–47), Thriving (48–56).

#### 2.5.6 Coping self-efficacy

The Coping Self-Efficacy Scale (CSES; Chesney et al., 2006) is a 13-item measure that assesses confidence to engage in specific coping strategies when under stress. Items on the CSES are rated on a 10-point Likert scale, ranging from 0 (*not at all confident*) to 10 (*completely confident*), with higher scores indicating greater coping self-efficacy. The scale has three factors assessing broad coping strategies: problem-focused coping (6 items, 0–60 range), stopping unpleasant thoughts and emotions (4 items, 0–40 range), and seeking support (3 items, 0–30 range). The 13-item measure was administered at the baseline and final assessments, while an abbreviated 3-item version (using highest factor-loading item from each of the three factors) was administered as part of the weekly Sunday surveys.

#### 2.5.7 Emotional awareness

The Difficulties with Emotion Regulation Scale (DERS; Gratz and Roemer, 2004) contains a six-item subscale that assesses emotional awareness, which was administered at baseline, weekly, and final assessments. Participants were asked to rate how often a series of statements apply to them using a 5-point Likert scale ranging from 1 (*almost never*) to 5 (*almost always*), with totals ranging from 6 to 30 and higher scores indicating greater emotional awareness. Sample items include: “I am attentive to my feelings,” and “When I'm upset, I acknowledge my emotions.”

#### 2.5.8 Loneliness

The UCLA Loneliness Scale (Hughes et al., 2004) is a three-item measure of loneliness, which was administered at baseline, weekly, and final assessments. Participants were asked to rate how often they have felt left out, isolated, and lacking companionship on a 3-point Likert scale ranging from 0 (*hardly ever*) to 2 (*often*), with totals ranging from 0 to 6 and higher scores indicating more loneliness.

#### 2.5.9 Mental health service utilization and readiness to seek help

One item from the Healthy Minds Study (Lipson et al., 2022) was used to assess history of mental health service utilization (“In your lifetime, have you received counseling or therapy for your mental or emotional health from a health professional (such as a psychiatrist, psychologist, social worker, or primary care doctor)?”). Follow-up items asked about past year and current use of services, when indicated. Four items assessing readiness for help-seeking from different sources (i.e., online resources, family/friends, professional services, support groups) were administered at baseline and final assessments using a 10-point scale (King et al., 2022; King et al., 2015), with two items (online resources, professional services) also administered at weekly surveys.

### 2.6 Data analytic plan

Descriptive statistics were used to report primary outcomes related to feasibility and acceptability, including percentages, means, standard deviations, medians, and interquartile ranges. A series of *t*-tests were conducted to examine equivalence at baseline for the intervention and assessment-only groups. Retention analyses utilized t-tests to compare those who completed the final follow-up assessment to those who did not complete the final assessment.

Exploratory analyses examined differences between conditions after six weeks via a series of ANCOVAs, with group assignment as an independent variable and controlling for baseline levels of the respective clinical outcome variables. Within-group comparisons consisted of mixed effect models examining whether proximal end-of-week clinical outcome scores differed among intervention group participants on weeks they received PF and/or supportive messages compared to weeks they did not, while controlling for prior-week levels of the outcome of interest. Mixed-effects models featured random intercepts (accounting for repeated measures) and random slopes for time (i.e., weeks in study). As a pilot trial, we were not formally powered for detecting effects with respect to clinical outcomes, and to reduce the risk of Type II errors, exploratory analyses did not include corrections for multiple testing.

## 3 Results

### 3.1 Study participants

The sample included first-semester undergraduate students (*N* = 120) with mild-to-moderate depressive symptoms who were not engaged in mental health treatment. A summary of the sample's demographic characteristics are presented in Table 1. Clinical characteristics at baseline and six-week follow-up are summarized in Table 2.

**Table 1:** Demographic characteristics (*N* = 120).

Category	N	%
Age		
17	16	13.3
18	90	75.0
19	12	10.0
20 or older	2	1.6
Gender Identity		
Woman	77	64.2
Man	38	31.7
Transgender or Non-Binary	5	4.2
Race and Ethnicity		
Non-Hispanic White	65	54.2
Asian	20	16.7
Multi-Racial	13	10.8
Hispanic or Latino	11	9.2
Black	9	7.5
Other	2	1.7
Sexual Orientation		
Heterosexual	82	68.3
Bisexual or Pansexual	14	11.7
Gay, Lesbian, Queer or Other	13	10.8
Mostly Heterosexual	11	9.2
Campus		
Flagship	104	86.7
Satellite	16	13.3

**Table 2:** Sample clinical characteristics.

	Full sample (N = 120)		Intervention (n = 90)		Assessment-only (n = 30)	
	Baseline (N = 120) *M*(SD)	6-week FU (*n* = 104) *M*(SD)	Baseline (*n* = 90) *M*(SD)	6-week FU (*n* = 78) *M*(SD)	Baseline (*n* = 30) *M*(SD)	6-week FU (*n* = 26) *M*(SD)
Depression	8.3 (2.7)	7.4 (4.9)	8.3 (2.6)	7.7 (4.7)	8.5 (3.2)	6.4 (5.4)
Anxiety	7.0 (4.5)	6.6 (4.8)	6.9 (4.5)	6.5 (4.8)	7.4 (4.5)	6.8 (4.8)
Perceived Stress	21.1 (5.1)	18.2 (5.7)	21.1 (5.5)	18.4 (5.7)	21.0 (3.9)	17.9 (5.7)
Flourishing	43.8 (6.4)	44.2 (7.3)	43.8 (6.5)	43.8 (7.5)	43.8 (6.3)	45.4 (6.7)
Emotional Awareness	20.4 (5.4)	20.7 (5.8)	20.9 (5.2)	20.9 (5.7)	18.9 (5.8)	20.2 (6.3)
Loneliness	2.7 (1.7)	2.2 (2.0)	2.9 (1.6)	2.7 (2.0)	2.0 (1.7)	2.1 (1.7)
Coping SE (Prob)	35.7 (10.0)	36.2 (11.4)	35.5 (9.6)	36.1 (10.8)	36.5 (11.3)	36.5 (13.2)
Coping SE (Emo)	18.8 (7.9)	21.5 (9.4)	18.7 (7.9)	21.4 (9.0)	19.3 (8.2)	21.6 (10.7)
Coping SE (Sup)	15.9 (6.9)	16.5 (7.5)	15.9 (6.7)	16.3 (7.6)	15.9 (7.4)	17.0 (7.4)
Help-seeking1	3.7 (3.0)	3.7 (3.2)	3.4 (3.0)	3.7 (3.2)	4.8 (2.9)	3.6 (3.4)
Help-seeking2	4.0 (3.3)	3.4 (3.1)	3.7 (3.1)	3.5 (3.2)	4.8 (3.8)	3.1 (3.0)
Help-seeking3	3.5 (3.0)	3.1 (3.0)	3.4 (3.0)	2.9 (3.0)	3.8 (3.0)	3.5 (3.2)
Help-seeking4	1.8 (2.1)	2.2 (2.7)	1.9 (2.2)	2.0 (2.6)	1.6 (1.8)	2.7 (3.1)

Note. *M* = Mean; *SD* = Standard Deviation; FU = Follow-Up; Coping SE = Coping Self-Efficacy; Emo = Stopping unpleasant thoughts/emotions; Sup = Seeking support; Prob = Problem-solving; Help-seeking1 = Readiness to seek information about mental health resources; Help-seeking2 = Readiness to talk to friends/family about possibly seeking help from mental health professional; Help-seeking3 = Readiness to seek help from a mental health professional; Help-seeking4 = Readiness to access self-help or support group.

### 3.2 Primary outcomes: feasibility and acceptability

A CONSORT Flow of screening, enrollment, and retention for the study is presented in Fig. 1. Results suggest a high degree of feasibility for trial enrollment and procedures, with 95.2% (120/126) of eligible participants proceeding to phase 2 of the study. Of the weekly Sunday surveys (delivered over five weeks to 120 participants), 85.5% (513/600) were completed. Additionally, 86.7% (104/120) participants completed the final six-week follow-up survey, with only two participants (1.7%) formally withdrawing during the study.

Intervention condition participants (*n* = 90) were sent an average of 13.53 (*SD* = 4.9) text messages and 4.03 (*SD* = 1.15) PF reports during the six-week intervention. The distribution of text messages received by students was as follows: 6.7% received 0–5 messages, 16.7% received 6–10 messages, 41.1% received 11–15 messages, 31.1% received 16–20 messages, and 4.4% received 21–25 messages. For PF reports: 10.0% received two or fewer reports, 21.1% received three reports, 32.2% received four reports, 27.8% received five reports, and 8.9% received six reports. Approximately 78% of participants viewed their PF webpage at least once, and 58.7% viewed multiple PF reports. The overall completion rate for daily evening surveys over six weeks was 69.2% (2614/3780).

Retention analyses were underpowered for significance testing due to only 16 participants being lost to follow-up. However, trends suggested that those who dropped out from the study experienced higher depression (9.56 vs. 8.14, *t*(18.4) = 1.72, *p* = .10) and anxiety (8.56 vs. 6.79, *t*(23.4) = 1.79, *p* = .09) scores at baseline compared to those who remained in the study.

With respect to acceptability, over 90% of participants indicated they were glad they participated in the study, viewed the messaging/assessment system as convenient, and felt their privacy/confidentiality was respected. Assessment procedures had the highest degree of support, with over 90% of participants agreeing that daily and weekly surveys helped them reflect on how things were going in their lives. Notably, fewer than 40% of participants indicated they would be willing to complete brief surveys (daily or weekly) without compensation. With respect to intervention components, 77% of participants believed that PF reports provided valuable insights, whereas 46% of participants believed the coping/supportive text message strategies were useful. A full summary of acceptability responses is presented in Table 3.

**Table 3:** Acceptability ratings for study and intervention components.

	*Median*	*IQR* (*Q1–Q3*)	% Agreement
1. I am glad that I participated in this study.^a^	6	6–7	93.3
2. The daily 5-item surveys helped me to reflect on how things were going in my life.	6	6–7	91.0
3. The coping/support text-message strategies were useful for me.	4	3–6	45.5
4. I would be willing to complete brief (∼5 min) weekly surveys during the year without compensation.^a^	4	2–5	37.3
5. The personalized feedback reports provided valuable insight into how I was doing over time.	5	5–6	76.6
6. The number of coping/support text messages I received was… (1—too few, 4—just right amount, 7—too many)	4	4–5	70.1⁎
7. Six weeks was too brief for this study period.^a^	4	3–5	37.3
8. Participating in this study was beneficial for my health and well-being.^a^	5	5–6	79.4
9. I would have liked to receive personalized feedback graphs every week.	5	4–6	57.1
10. The weekly Sunday surveys helped me to reflect on how things were going in my life.^a^	6	5–6	90.2
11. I would be willing to complete brief (∼1 min) daily surveys during the year without compensation.	4	3–5	37.7
12. The study's intervention meets my approval.	6	5–6	76.6
13. The system of receiving messages and completing surveys was convenient.^a^	6	6–7	94.1
14. The study's intervention was appealing to me.	6	5–6	79.2
15. A program like this study (e.g., regular surveys, text messages, and personalized feedback reports) should be offered to all students.	6	5–6	85.7
16. I would want to continue receiving surveys, texts, and personalized feedback reports after this study is complete.	4	3–5	42.9
17. I like the intervention from this study.	5	4–6	67.5
18. My confidentiality and privacy was respected during this study.^a^	6	6–7	90.2

*Note*. *n* = 77. Mdn = Median. IQR = Interquartile Range. All items were rated on a 1–7 scale ranging from 1 (*strongly disagree*) to 7 (*strongly agree*) with 4 as *neither agree nor disagree*. % Agreement = Slightly agree (5) or higher.

a Indicates item administered to both intervention and assessment-only groups with total n of 102.

⁎ % Agreement for this item was based on responding with a 3, 4, or 5.

### 3.3 Exploratory analyses: clinical outcomes

Despite anticipation of increased symptom severity over the course of the study, clinical symptoms slightly improved for both groups (see Table 2). In assessing equivalence based on group randomization, several baseline differences emerged, including significantly greater loneliness (*M* = 2.88 vs. 1.97, *t*(47.3) = 2.53, *p* = .014) and significantly lower readiness to seek information about available mental health resources (*M* = 3.40 vs. 4.77, *t*(50.9) = 2.22, *p* = .031) among the intervention group.

A series of one-way, between-group ANCOVAs did not reveal significant differences in outcomes based on group randomization, with a lone exception— after controlling for baseline readiness, assessment-only participants had significantly lower adjusted follow-up readiness scores to talk to family/friends about possibly seeking professional mental health services compared to the intervention group (b = −1.21, SE = 0.59, *t*(101) = 2.05, *p* = .043; Estimated marginal means (SE) = 2.47 (0.51) vs. 3.68 (0.29)). Initiation of psychotherapy services during the follow-up period did not significantly differ between the intervention (*n* = 5, 6.4%) and assessment-only (*n* = 2, 7.7%) groups.

Mixed-effects models assessing the weekly effects of intervention exposure (PF and/or supportive text messages) on clinical outcomes indicated a slight reduction in readiness to seek online mental health resources during weeks in which text messages were received. However, overall trends did not suggest meaningful end-of-week clinical differences based on receiving or not receiving intervention components (see Table 4).

**Table 4:** Analysis of micro-randomizations to interventions on end-of-week outcomes.

	B (95% CI)	SE	t
Depression			
Intercept	4.88 (3.75, 6.00)	0.57	8.51⁎⁎⁎
Texts	0.21 (−0.28, 0.71)	0.25	0.84
PF	0.15 (−0.38, 0.67)	0.27	0.54
Week	0.18 (0.03, 0.32)	0.08	2.31⁎
Lagged_Dep	0.21 (0.12, 0.30)	0.05	4.68⁎⁎⁎
Anxiety			
Intercept	4.98 (3.88, 6.07)	0.56	8.91⁎⁎⁎
Texts	0.22 (−0.28, 0.73)	0.26	0.86
PF	−0.26 (−0.80, 0.27)	0.27	−0.96
Week	0.03 (−0.12, 0.17)	0.07	0.36
Lagged_Anx	0.24 (0.15, 0.32)	0.04	5.39⁎⁎⁎
Stress			
Intercept	4.90 (4.04, 5.77)	0.44	11.14⁎⁎⁎
Texts	0.18 (−0.15, 0.51)	0.17	1.05
PF	0.08 (−0.28, 0.43)	0.18	0.42
Week	0.00 (−0.10, 0.11)	0.05	0.03
Lagged_Stress	0.28 (0.20, 0.36)	0.04	6.68⁎⁎⁎
Flourishing			
Intercept	30.09 (26.36, 33.81)	1.90	15.82⁎⁎⁎
Texts	−0.23 (−0.90, 0.45)	0.35	−0.66
PF	0.14 (−0.57, 0.85)	0.36	0.38
Week	0.07 (−0.12, 0.26)	0.09	0.74
Lagged_Flourish	0.30 (0.22, 0.38)	0.04	7.38⁎⁎⁎
Emo_Aware			
Intercept	15.09 (13.02, 17.16)	1.06	14.27⁎⁎⁎
Texts	−0.15 (−0.63, 0.32)	0.24	−0.63
PF	0.32 (−0.18, 0.82)	0.26	1.24
Week	0.18 (0.05, 0.30)	0.06	2.75⁎
Lagged_Aware	0.22 (0.14, 0.30)	0.04	5.15⁎⁎⁎
Help-seeking1			
Intercept	3.94 (3.19, 4.68)	0.38	10.36⁎⁎⁎
Texts	−0.28 (−0.55, −0.01)	0.14	−2.05⁎
PF	−0.06 (−0.35, 0.23)	0.15	−0.38
Week	0.06 (−0.06, 0.18)	0.06	0.99
Lagged_help1	−0.12 (−0.20, −0.04)	0.04	−2.88⁎⁎
Help-seeking3			
Intercept	2.84 (2.17, 3.52)	0.34	8.25⁎⁎⁎
Texts	0.14 (−0.13, 0.42)	0.14	1.05
PF	0.02 (−0.27, 0.31)	0.15	0.12
Week	−0.03 (−0.13, 0.08)	0.05	−0.48
Lagged_help3	0.05 (−0.03, 0.13)	0.04	1.18

Note. Texts = Randomized to receive text messages that week (0 = No, 1 = Yes). PF = Randomized to receive a personalized feedback report that week (0 = No, 1 = Yes). Emo_Aware = Emotional Awareness. Help-seeking1 = Readiness to seek help from online resources. Help-seeking3 = Readiness to seek help from a mental health professional. Models were based on 460 observations from 83 participants. All models included random intercepts and random slopes for time (week) at the participant level. Each model included a lagged variable controlling for the prior week levels of the dependent variable.

⁎ *p* < .05.

⁎⁎ *p* < .01.

⁎⁎⁎ *p* < .001.

## 4 Discussion

This pilot study examined the feasibility and acceptability of a low-burden digital mental health intervention (DMHI) incorporating regular assessments, personalized feedback (PF), and supportive text messages over six weeks for first semester undergraduate students with mild-to-moderate depressive symptoms. Feasibility was evidenced by strong adherence to weekly surveys, high enrollment rates among those eligible for the clinical trial, and overall retention at the final assessment. Acceptability ratings for the overall intervention were very favorable, with particularly positive ratings for the helpfulness of daily and weekly surveys. The PF component of the intervention was viewed as helpful by 76% of participants, whereas slightly less than half agreed that the supportive text messages were helpful.

There are several possible explanations for the lack of enthusiasm for supportive text messages. Given that the supportive text messages were delivered at random intervals, there may have been mismatches between timing and content (i.e., receiving a text message about coping with stress on a day when they were not stressed) which reduced their perceived benefit. Thus, tailoring message content based on student preferences (e.g., frequency, timing) or context cues (e.g., targeting specified area of concern such as sleep or anxiety) may increase perceived helpfulness and utility of these messages. Further, there may have been less perceived need for supportive messages, particularly because most participants experienced only minimal-to-mild depressive symptoms during the study period. In contrast, receiving PF reports in the context of minimal-to-mild symptoms may have been less likely to elicit negative reactions, possibly due to the limited frequency of reports (i.e., no more than once per week) or because the content was tailored to reflect their minimal symptom status. Further analyses are needed to determine whether acceptability may have been moderated by sociodemographic or clinical factors (e.g., severity of anxiety/depression, prior use of mental health services), which could inform the development of an adaptive intervention approach that optimizes timing and content for supportive texts and PF reports. If so, it may be that PF is an acceptable intervention at milder symptom levels, while supportive text messages are more appropriate when depressive symptoms are elevated.

Interestingly, between- and within-group analyses did not suggest improvement in clinical outcomes despite participants' favorable view of the overall intervention. Specifically, between-group comparisons examining the intervention and assessment-only conditions did not reveal significant differences in clinical outcomes, with the exception that those in the intervention condition had less change in readiness to speak to family/friends about seeking help than the assessment-only condition. Notably, both groups demonstrated slight improvements in clinical outcomes over the course of six weeks, which contrasts with intensive longitudinal studies demonstrating increases in stress and decreases in positive affect over time (e.g., Garett et al., 2017; Rogers et al., 2018). There may be differences in exact times that stress peaks across schools based on semester vs. quarter structures or seasonal factors based on geography. It may also be that the present study enrolled students when symptoms were already starting to peak (early October, over one month into the semester) and concluded (mid-late November) prior to a final increase in stress associated with finals. Despite randomization, there were between-group differences at baseline (i.e., intervention group was more lonely and less ready to seek online information about mental health resources), further challenging between-group comparisons with limited power. With respect to lack of findings for within-group comparisons, the anticipated modest effects of a light-touch intervention may have been masked by unique environmental factors (e.g., fall break, midterms, presidential election, etc.) that played a greater role in week-to-week variation in stress and symptoms.

Various studies have indicated that self-monitoring alone can improve mental health symptoms (e.g., Chen et al., 2017; Kauer et al., 2012), even when compared to more active interventions (e.g., Watkins et al., 2024), suggesting the weekly surveys may have benefited the assessment-only group. While our assessment-only condition did not provide any explicit instructions with respect to self-monitoring, the weekly process of completing the same questions (and seeing links to available resources) may represent an even lower burden approach to this intervention—comparing a weekly assessment condition to a pre/post-only assessment may clarify potential benefits. This improvement, combined with the restricted variance in symptom severity (due to screening process), may have limited the amount of symptom change that could have been detected compared to trials for participants with more severe depressive symptoms. Even so, within-group comparisons examining weekly variations in clinical outcomes based on the presence or absence of the intervention components (supportive texts and/or PF) did not suggest meaningful differences between intervention and non-intervention weeks. A notable exception was that the proximal effect of receiving supportive text messages was associated with reduced readiness to seek out online mental health resources that week. However, it is unclear whether this reduced readiness might reflect less perceived need due to already receiving the supportive text messages or, conversely, having a dislike for the supportive messages that reduces interest in online resources. Nevertheless, similar studies have demonstrated high levels of perceived helpfulness despite limited clinical efficacy (Folkersma et al., 2021), suggesting symptom outcomes may not necessarily capture full intervention value.

### 4.1 Limitations

The findings from this study should be interpreted within the context of its limitations. While our study included students from both the flagship and satellite campuses, these campuses are not nationally representative of first-year university students, thus findings may not generalize to other students or campuses. Our study included objective data regarding participants viewing the PF reports but did not have similar data about whether supportive text messages were read (versus ignored or missed), which limits insight into potential mechanistic links of engagement with the intervention content on outcomes. Our consent rate among e-mailed participants (14%) was in line with expectations, yet there may have been selection biases with respect to the types of students who were interested in participating—acceptability of these interventions and procedures might differ among those who did not wish to participate. Importantly, as a pilot feasibility study, we were not formally powered to assess clinical efficacy, and there were several features (e.g., non-equivalency between the two conditions at baseline, mild symptom profiles, potential benefits associated with the assessment-only condition) that further limited our exploratory analyses. Screening for inclusion at a single time point may have resulted in including participants who were randomly above their expected depression mean at that time, and whose improvements may have been attributable to regression to the mean. To reduce this risk of bias in the future, it may be appropriate to screen for symptom elevation for consecutive weeks prior to enrollment, and to consider different timepoints across the semester or school year to examine symptom changes. Finally, participants were compensated for completing assessments and indicated on acceptability measures that they would be less inclined to engage without financial incentive. Therefore, universities may need to identify and develop creative ways to incentivize survey completion if self-report assessments were regularly administered as part of a campus health program.

### 4.2 Conclusions and future directions

This pilot feasibility trial investigated the use of a low-burden DMHI as a secondary prevention strategy for first-year college students with mild-to-moderate depressive symptoms. Findings suggest feasibility and acceptability for a system of assessment/self-monitoring, personalized feedback, and supportive text messages, and presents opportunities to improve the acceptability of supportive text messages. There were no consistent within-group or between-group patterns to suggest an intervention effect, though this may have been due to limited statistical power, mild symptom profile of the sample, and potential benefits from self-monitoring across groups. Even so, the sustained rates of participation and high favorability ratings suggest that this system may represent a scalable and engaging first step that can be expanded into an adaptive intervention that incorporates higher-intensity DMHIs, human-delivered sessions, and/or connections to mental health resources in response to needs over time. Additional research with larger samples and more diverse campus settings is needed to better tailor content, timing, and intensity of these DMHIs, and to identify opportunities for integration within existing campus mental health support systems.

## Declaration of competing interest

The authors declare that they have no known competing financial interests or personal relationships that could have appeared to influence the work reported in this paper.
